# Impact of Transcranial Magnetic Stimulation on Functional Movement Disorders: Cortical Modulation or a Behavioral Effect?

**DOI:** 10.3389/fneur.2017.00338

**Published:** 2017-07-19

**Authors:** Béatrice Garcin, Francine Mesrati, Cécile Hubsch, Thomas Mauras, Iulia Iliescu, Lionel Naccache, Marie Vidailhet, Emmanuel Roze, Bertrand Degos

**Affiliations:** ^1^Neurology Department, Parkinson’s Disease Expert Centre, Pitié-Salpêtrière Hospital, AP-HP, Paris, France; ^2^Institut du Cerveau et de la Moelle épinière (ICM), UPMC UMRS 1127, INSERM U 1127, CNRS UMR 7225, Paris, France; ^3^Neurophysiology Department, Pitié-Salpêtrière Hospital, AP-HP, Paris, France; ^4^Psychiatry Department, Pitié-Salpêtrière Hospital, AP-HP, Paris, France; ^5^CNRS-UMR 7241/INSERM U1050, CIRB, Collège de France, UPMC, Paris, France

**Keywords:** functional movement disorders, treatment, transcranial magnetic stimulation, suggestion, neuromodulation, psychogenic

## Abstract

**Introduction:**

Recent studies suggest that repeated transcranial magnetic stimulation (TMS) improves functional movement disorders (FMDs), but the underlying mechanisms are unclear. The objective was to determine whether the beneficial action of TMS in patients with FMDs is due to cortical neuromodulation or rather to a cognitive-behavioral effect.

**Method:**

Consecutive patients with FMDs underwent repeated low-frequency (0.25 Hz) magnetic stimulation over the cortex contralateral to the symptoms or over the spinal roots [root magnetic stimulation (RMS)] homolateral to the symptoms. The patients were randomized into two groups: group 1 received RMS on day 1 and TMS on day 2, while group 2 received the same treatments in reverse order. We blindly assessed the severity of movement disorders before and after each stimulation session.

**Results:**

We studied 33 patients with FMDs (dystonia, tremor, myoclonus, Parkinsonism, or stereotypies). The median symptom duration was 2.9 years. The magnetic stimulation sessions led to a significant improvement (>50%) in 22 patients (66%). We found no difference between TMS and RMS.

**Conclusion:**

We suggest that the therapeutic benefit of TMS in patients with FMDs is due more to a cognitive-behavioral effect than to cortical neuromodulation.

## Introduction

Individuals with functional movement disorders (FMDs) account for 3–20% of all patients seen in movement-disorder clinics (1–3). There is no consensus treatment for FMDs (4–6). These movement disorders are not due to irreversible brain damage but their outcome is nonetheless poor: symptoms are persistent or worse after 1.5–7 years of follow-up in between 44 and 90% of patients (6, 7). FMDs generate major healthcare costs, as well as indirect costs due to unemployment and disability (8).

Recent studies suggest a beneficial effect of repeated supraliminal low-frequency transcranial magnetic stimulation (TMS) (i.e., TMS ≤ 1 Hz) on functional motor symptoms (9–14) [Ref. (15) for a review]. Among these studies, only one included a blinded assessment (11), and only one included a control group (sham treatment) (9). Focusing on FMDs more specifically, two studies showed a beneficial effect of supraliminal low-frequency TMS, with a mean improvement rate of 67% (11) and 97% (13). It is unclear whether the therapeutic benefit is due to cortical neuromodulation, i.e., to changes in cortical excitability and in connectivity between brain areas (15, 16). The alternative hypothesis is a cognitive-behavioral effect, a therapeutic effect that is linked to suggestion and/or motor relearning.

To address this issue, we blindly compared the therapeutic effect of repeated TMS and repeated root magnetic stimulation (RMS) in patients with FMDs. RMS was chosen as the control treatment to mimic TMS-induced movement without directly stimulating the cortex.

## Materials and Methods

### Patient Population

Patients were eligible if they were 18 years or older and fulfilled the clinical criteria for FMD as defined by Fahn and Williams (17) (Table 1). Patients were excluded if they had a history of other neurological disorder or psychosis; if another neurological disease was diagnosed during hospitalization for magnetic stimulation; if they had received TMS in the past; or if they had a contraindication to magnetic stimulation. We enrolled consecutive FMD inpatients seen in our Movement Disorders Clinic between April 2013 and July 2015. The study was approved by the local ethics committee (CPP-IdF-Paris 6, Pitié-Salpêtrière University Hospital), and all the patients gave their informed consent.

**Table 1:** Description of the patients.

	Patients (*n* = 33)
Gender: male [*n* (%)]	7 (21.2)
Age [median (IQR)]	45 (28.6–54.9)
Education in years [median (IQR)]	12 (10–15)
Work or study^a^ [*n* (%)]	12 (36.4)
Symptom duration in years [median (IQR)]	2.9 (1.6–10.5)
Clinical presentation	
Tremor [*n* (%)]	13 (39.4)
Dystonia [*n* (%)]	11 (33.3)
Jerky dystonia [*n* (%)]	4 (12.1)
Myoclonus [*n* (%)]	2 (6.1)
Stereotypies [*n* (%)]	2 (6.1)
Parkinsonism [*n* (%)]	1 (3)
Depression and/or anxiety [*n* (%)]	20 (60.6)
Traumatic life events [*n* (%)]	21 (63.6)
Sexual abuse [*n* (%)]	8 (24.2)
Other major trauma [*n* (%)]	13 (39.4)
Hospital Anxiety and Depression total score [median (IQR)]	11 (7–17.5)
FMD score at baseline [median (IQR)]	19 (14–24)
Improvement after session 1 [% (IQR)]	29.2 (11.8–60)
Improvement after session 2 [% (IQR)]	18.2 (0–44)
Total improvement at day 3 [% (IQR)]	70 (27–100)
Patients who relapsed^b^ [*n* (%)]	12 (36.4)

*^a^The other patients had quit their studies, were unemployed, retired on long-term sick leave, or on disability living allowance*.

*^b^Relapses that occurred within 1 year follow-up for 32 patients, or within 6 months follow-up for the remaining patient*.

*IQR, interquartile range*.

### Study Design

**Figure 1:**
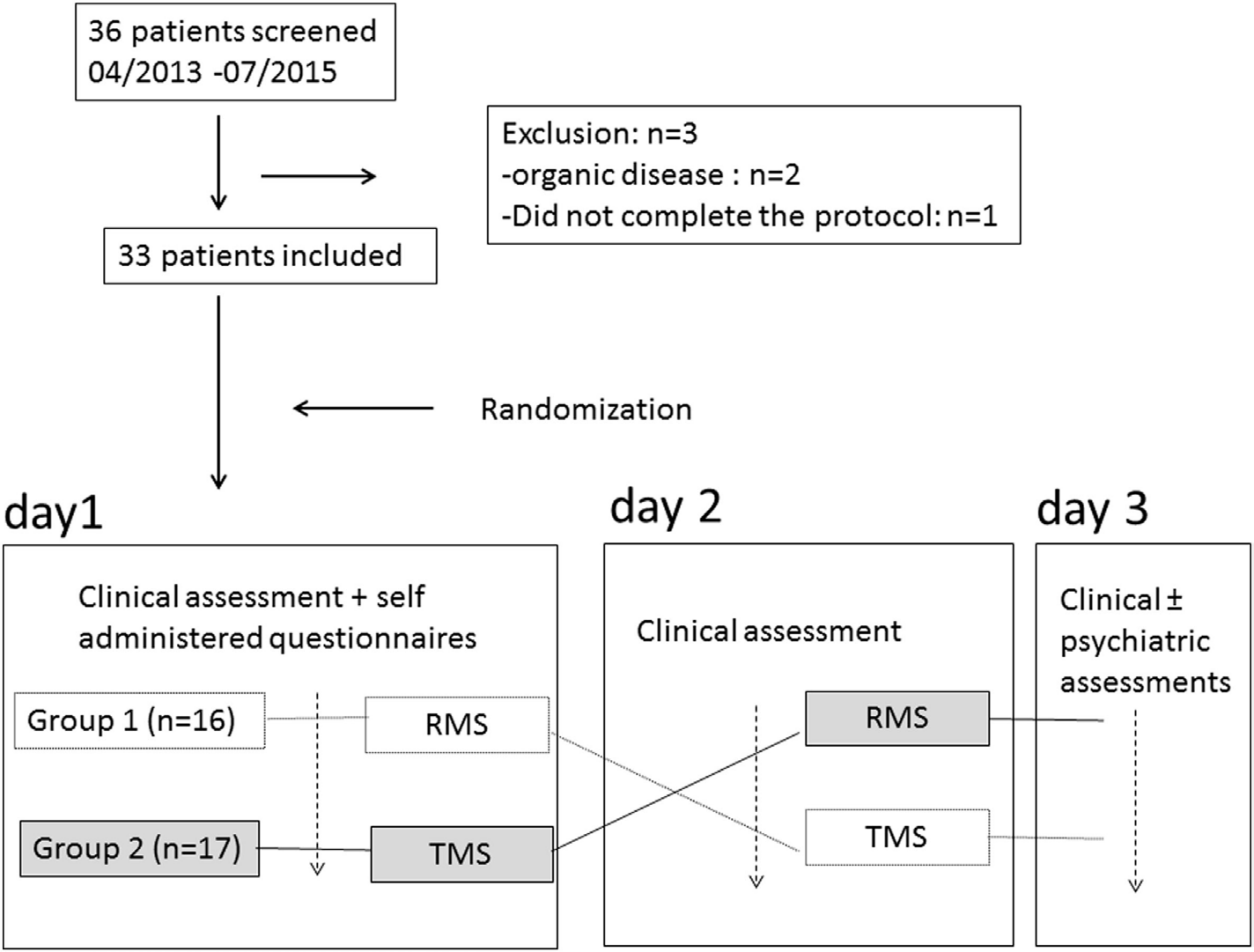
Study design. RMS, root magnetic stimulation; TMS, transcranial magnetic stimulation.

The patients were informed that their symptoms were linked to a non-lesional brain dysfunction (Figure 1). The patients were prospectively randomized to receive RMS on day 1 and TMS on day 2 (group 1) or the reverse sequence (group 2). A randomization function was used in Excel (Microsoft Excel RAND function) to establish the order of magnetic stimulations before the beginning of the study. A minimal interval of 18 h was respected between the two treatment sessions.

### Neurological Assessment

A detailed clinical assessment was done by CH on day 1 (baseline score, before stimulation 1), day 2 (before stimulation 2), and day 3 (after stimulation 2). Each clinical assessment was videorecorded, and one movement-disorder expert (BD) blindly rated the patients before and after each session, using a rating scale that was specifically designed for FMDs (11) (Data Sheet 1 in Supplementary Material), yielding an “FMD score” ranging from 7 (normal) to 41 (most severe). The percentage of improvement was calculated as follows: ((pretreatment score) − (posttreatment score))/((baseline score) − 7). No other treatment was provided during the hospital stay, and the patients’ medication was kept stable during the study. Physiotherapy was not provided during the protocol, but it was systematically prescribed at discharge. A new TMS session was performed when patients relapsed during the follow-up. No psychotherapy was offered during the whole duration of follow-up.

### Psychiatric Assessment

On day 1, all the patients completed self-administered psychiatric questionnaires, including a screening version of the Mini International Neuropsychiatric Interview (MINI) (18), the Hospital Anxiety and Depression (HAD) Scale (19), and the exposure to traumatic life events section of the French version of the Composite International Diagnostic Interview (CIDI 2.1) (20). These evaluations allowed us to explore mood and anxiety disorders, addictive behaviors, post-traumatic disorders, and psychotic disorders. When a psychiatric examination was considered necessary, because of a positive MINI section, a behavioral disorder, or the care team opinion, a psychiatrist (Thomas Mauras) interviewed the patient on day 3 (*n* = 16, 48.5%) or in the psychiatric clinic after hospital discharge (*n* = 6, 18.1%).

### Information Given to Patients

At inclusion, magnetic stimulation was described to the patients as an effective treatment with excellent results. However, to ensure a reproducible protocol, the neurophysiologist (Iulia Iliescu or Francine Mesrati) said nothing to reinforce perceived treatment efficacy during the stimulation session.

### Magnetic Stimulation

An average of 50 (range: 30–80) consecutive stimuli, at 120–150% of the resting motor threshold (each stimulus lasted 250 µs) was delivered at low frequency (0.25 Hz) over two different sites, namely, the lateral (upper limbs) or medial (lower limbs) motor cortex contralateral to the FMD for repeated TMS, and over the cervical (upper limbs) or lumbar (lower limbs) spinal roots homolateral to the FMD for repeated RMS. The raters (Bertrand Degos and Cécile Hubsch) were blinded to the type of stimulation.

### Follow-up

The patients were followed up with telephone interviews at 3 and 12 months (Cécile Hubsch) and with a visit (Bertrand Degos or Cécile Hubsch) 6 months after treatment. The Clinical Global Impression—Improvement (CGI-I) Scale (21) was estimated by the patients at each follow-up. Patients were asked how the severity of movement disorder was as compared to pretreatment. This scale ranges from 1 (very much improved) to 7 (very much worse).

### Statistical Analysis

SPSS software (http://www-01.ibm.com/software/analytics/spss/) was used for all analyses, and significance was assumed at *p* < 0.05. The distribution of the percentage improvements did not follow a normal distribution (Shapiro–Wilk test, *p* = 0.002 for global improvement). For this reason, and because of the small number of patients in each group, we used non-parametric tests. Spearman’s rank correlation test was used for correlation analyses. The Wilcoxon signed-rank test was used for group comparisons and the Kruskal–Wallis test was used to compare scores across more than two groups. A mixed three-factor ANOVA was used to determine the effect of (i) the type of stimulation (root versus transcranial), (ii) the day of stimulation (day 1 or day 2), and (iii) the intervention group (group 1 or 2). The condition of a normal distribution of residuals was respected; this allowed us to use an ANOVA for this multifactorial analysis.

## Results

### Patient Characteristics

Thirty-six consecutive patients with FMDs participated in the study (Table 1). Two patients were excluded because of an “organic” condition (Parkinson’s disease and algoneurodystrophy syndrome), and one patient was excluded because he refused magnetic stimulation. The remaining 33 patients (26 F/7 M) were included in the analyses. Their main characteristics are summarized in Table 1, and a detailed description is provided in Table S1 in Supplementary Material.

Median age was 45 years (range: 18–74). The median symptom duration was 2.9 years (range: 0.3–30 years). The predominant movement disorders were tremor (*n* = 13), dystonia (*n* = 11,), jerky dystonia (*n* = 4), myoclonus (*n* = 2), stereotypies (*n* = 2), and parkinsonism (*n* = 1). Associated disorders included motor deficits (*n* = 7; 21.2%), sensory deficits (*n* = 7; 21.2%), and pain (*n* = 8; 24.2%).

Ongoing anxiety disorders were found in 20 patients (60.6%) and included generalized anxiety disorder, panic disorder, social phobia, agoraphobia, and obsessive–compulsive disorder. The anxiety disorder was associated with depression in seven patients (21.2%). Twenty-one patients (63.6%) reported a traumatic life event according to the traumatic life events section of the French version of the CIDI 2.1, including rape in 24.2% of cases and another life-endangering trauma in 39.4%. Only 12 patients (36%) were in employment or study, the remaining 21 patients (64%) being on long-term sick leave (with a disability living allowance), unemployed, retired, or students having interrupted their studies. Fifteen of the latter 21 patients were receiving disability-related benefits.

### Improvement after Magnetic Stimulation: Whole Population

**Figure 2:**
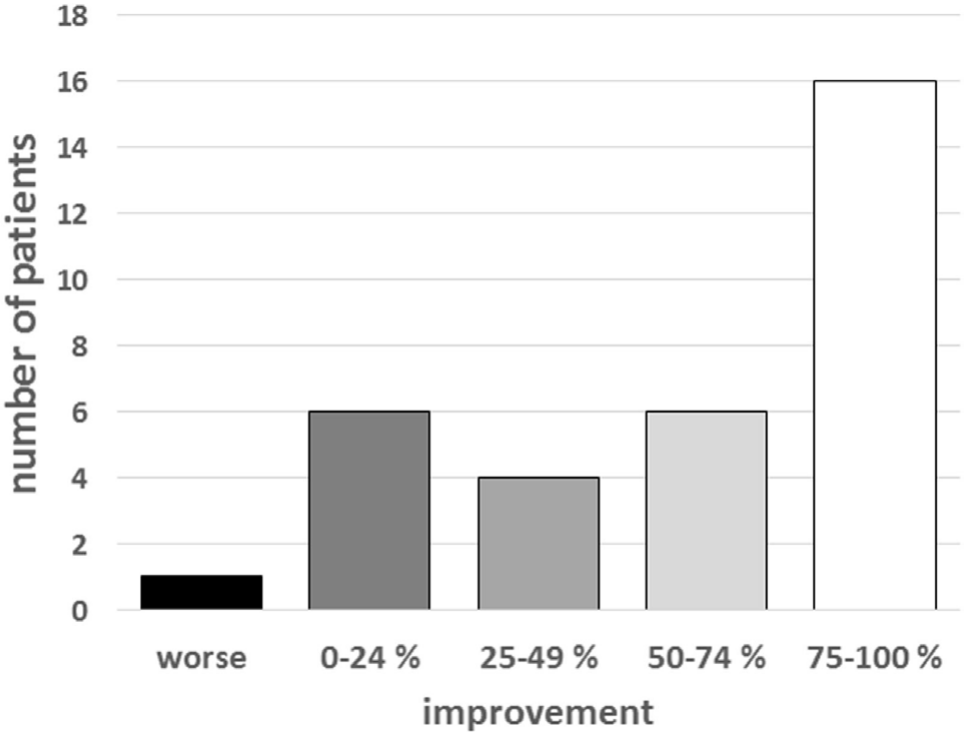
Patient distribution according to the degree of improvement on day 3. The chart represents the numbers of patients who were improved by >75, 50–75, 25–50, and <25%.

The median percentage improvement in FMD scores was 29.2% after the first session and 18.2% after the second session (Table 1). The median total percentage improvement was 70% at day 3. Twenty-two patients (66.7%) experienced a significant improvement (>50% improvement) (Figure 2). Motor symptoms resolved completely on day 3 in 10 of these latter patients (30%).

We found no correlation between the final percentage improvement and age (*r* = 0.018, *p* = 0.92), gender (*z* = −1.095, *p* = 0.27), education (*r* = −0.004, *p* = 0.98), HAD scores (*r* = −0.134, *p* = 0.45), depression and/or anxiety (*z* = −0.972, *p* = 0.33), traumatic life events (*z* = −1.747, *p* = 0.08), symptom duration (*r* = 0.315, *p* = 0.74), clinical presentation (Kruskal–Wallis: 2.8, *p* = 0.24, Table S2 in Supplementary Material), presence of any additional symptoms (deficit and/or pain) (*z* = −0.26, *p* = 0.811), or initial severity (*r* = 0.11, *p* = 0.53).

### Comparison of the Intervention Groups

Patients in group 1 (*n* = 16) and group 2 (*n* = 17) did not differ significantly with respect to age (*z* = −0.20, *p* = 0.84), gender (Fisher’s test, *p* = 0.4), symptom duration (*z* = −0.13, *p* = 0.9), clinical presentation (χ^2^ = 8.3, *p* = 0.19), or FMD severity (*z* = 0.81; *p* = 0.42) (Table 2).

**Table 2:** Comparison of the intervention groups.

	Group		
	Root stimulation first	Transcranial stimulation first	
Number of patients	16	17	
Age [median (IQR)]	43.8 (24.1–57.2)	46.1 (28.9–54.9)	Wilcoxon, *p* = 0.84
Gender: male [*n* (%)]	2 (12.5)	4 (23.5)	Fisher, *p* = 0.4
Education in years [median (IQR)]	12.5 (9–12.9)	12 (10–15)	Wilcoxon, *p* = 1
Symptom duration in years [median (IQR)]	3.4 (1.4–12.9)	2.8 (2.3–10.5)	Wilcoxon, *p* = 0.90
Depression and/or anxiety [*n* (%)]	13 (81.25)	12 (70.6)	Fisher, *p* = 0.38
Clinical presentation			Chi-square, *p* = 0.14
Tremor [*n* (%)]	6 (37.5)	7 (41.2)	
Jerky dystonia [*n* (%)]	3 (18.8)	1 (5.9)	
Dystonia [*n* (%)]	3 (18.8)	8 (47.1)	
Myoclonus [*n* (%)]	2 (12.5)	0 (0)	
Stereotypies [*n* (%)]	2 (12.5)	0 (0)	
Parkinsonism [*n* (%)]	0 (0)	1 (5.9)	
Baseline score [median (IQR)]	20.5 (16–29)	19 (12–24)	Wilcoxon, *p* = 0.42
% Improvement after first session [median (IQR)]	23.6 (5.5–47.8)	37.5 (20–72.7)	Wilcoxon, *p* = 0.29
Patients improved >50% after first session [*n* (%)]	4 (25)	8 (47)	Chi-square, *p* = 0.17
Total% improvement at day 3 [median (IQR)]	79.6 (24.7–100)	66.7 (50–100)	Wilcoxon, *p* = 1
Patients improved by >50% [*n* (%)] at day 3	9 (56.3)	13 (76.5)	Chi-square, *p* = 0.21

*IQR, interquartile range*.

There was no significant difference between the improvements noted after the first session of repeated TMS (median: 37.5%; IQR: 20–72.7) and after the first session of repeated RMS (median: 23.6%; IQR: 5.5–47.8) (*z* = −1.05; *p* = 0.29, Wilcoxon signed-rank test).

There was no significant difference in the final percentage improvement (day 3) between the two groups (*z* = 0; *p* = 1).

### Multifactorial ANOVA

There was no significant effect of the treatment modality (median improvement 35.8% after TMS versus 24.8% after RMS, *p* = 0.26), and no significant effect of the treatment order (RMS first or TMS first, *p* = 0.8) (Table 3). Regardless of the sequence, the percentage improvement was significantly larger after the first magnetic stimulation session (day 1) than after the second session (day 2) (*p* = 0.03).

**Table 3:** Multifactorial ANOVA.

Factors	% Improvement, mean (median) ± SD	
**Treatment modality**		
Root magnetic stimulation (RMS)	24.8 (20) ± 38.9	*p* = 0.26
Transcranial magnetic stimulation (TMS)	35.8 (33) ± 37.8	
**Order of treatment (final improvement)**		
RMS first	58.3 (79.6) ± 45.8	*p* = 0.8
TMS first	62.8 (67) ± 40	
**Day of treatment**		
After day 1	40.7 (29.2) ± 36.9	*p* = 0.03*
After day 2	19.9 (28.3) ± 37.8	

*p <0.05.

### Follow-up

**Figure 3:**
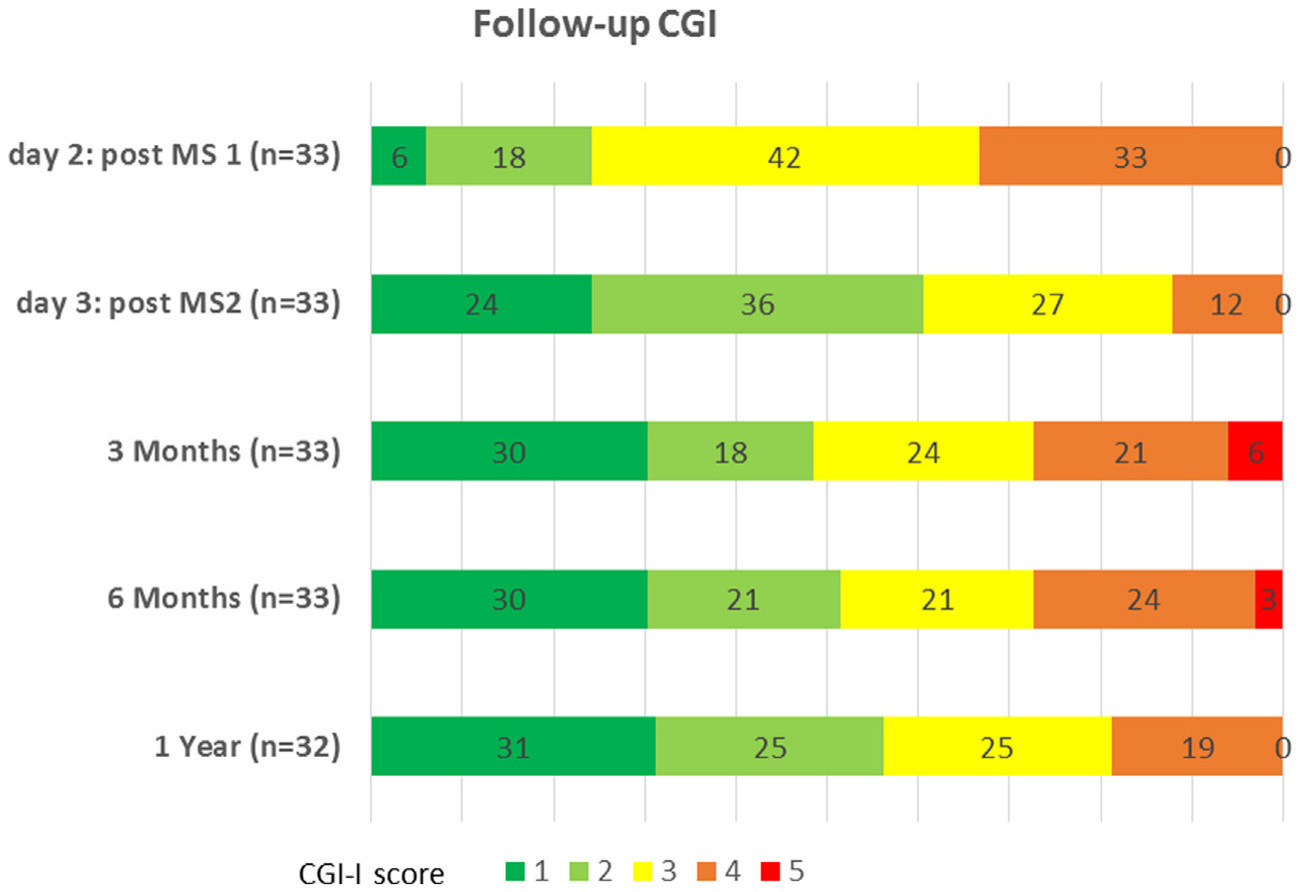
Follow-up Clinical Global Impression—Improvement (CGI-I scale). The chart represents the % of patients in each CGI-I score group. A CGI-I score is defined as follows: 1: very much improved; 2: much improved; 3: minimally improved; 4: no change; and 5: minimally worse.

All the patients were followed up for 6 months, and 29 patients (87.9%) were followed up for 1 year (Figure 3). On day 3, 24 h after the second session of magnetic stimulation, 60% of the patients were much (CGI score = 2) or very much (CGI score = 1) improved. At 1 year, 56% of the patients were still much or very much improved. Chi-square analysis revealed no statistical difference between the CGI score on day 3 and the CGI score at 3 months, 6 months, or 1 year (*p* = 0.77). However, 12 patients relapsed (total number of relapses = 19, median time to first relapse = 6 months). All the patients concerned were offered another TMS session at least 3 months after the previous session, and all were significantly improved after each relapse by another session of repeated TMS. These additional TMS sessions obviously influenced the follow-up CGI assessment at 6 months and 1 year.

## Discussion

We found no significant differences between the therapeutic efficacy of root and TMS in patients with FMDs, suggesting that magnetic stimulation acts mainly through a cognitive-behavioral effect rather than by cortical neuromodulation.

The degree of improvement did not differ between a first session of repeated TMS and a first session of repeated RMS. For a more powerful comparison, we performed a cross-over analysis taking all the sessions into account. Again, we found no difference between the two modalities of stimulation. These results suggest that TMS does not have a neuromodulatory effect on cortical functioning. This is in keeping with physiological studies showing that low-frequency (below 1 Hz) TMS does not induce changes in cortical excitability and therefore has no long-lasting neuromodulatory effects (22). Moreover, it is unlikely that the beneficial effects of TMS are mediated by changes in cortical excitability, as a wide range of TMS settings have been reported to improve functional neurological symptoms (15). Finally, the durability of the therapeutic effect of TMS observed here was unexpected, in view of previous reports of TMS neuromodulation in neurological disorders ((23) for a review). We propose that the therapeutic efficacy of TMS in patients with FMDs is mainly due to a cognitive-behavioral effect rather than to genuine neuromodulation. This cognitive-behavioral effect could occur through two main mechanisms, namely motor relearning and suggestion (15, 24). It is noteworthy that all TMS protocols that proved efficient in FMDs have used intensities of stimulation that were above motor threshold (15). Only one study used TMS at a subthreshold intensity (90% of motor threshold) in six patients with FMDs, and failed to show any efficacy (10). Suprathreshold intensity of TMS might thus be an essential prerequisite for efficacy. During suprathreshold magnetic stimulation sessions, patients experience unexpected stimulation-induced movement of their affected limbs; this may make the patient realize that his or her motor system is working properly and thereby allow the brain to “relearn” or “reprogram” a normal pattern of movement (25, 26). Comparing suprathreshold and subthreshold intensities of stimulation would be particularly interesting to underpin this hypothesis. Altered sense of agency due to a lack of feedforward signals may be critical in the pathogenesis of functional neurological disorders (27). Generating involuntary muscle twitches with suprathreshold magnetic stimulation may promote restoration of a normal sense of agency. There might also be an effect of suggestion, possibly linked to expectations of remission. Indeed, before they were treated, we informed the patients that magnetic stimulation was highly effective (11, 28). A broad range of therapeutic approaches, including acupuncture, hypnosis, and various drugs have occasionally been reported to be effective on FMDs [Ref. (5, 29) for a review]. This means that a single mechanism whereby brain activity is restored and symptoms relieved is highly improbable; rather, these different approaches are all more likely to act through suggestion or, in other words a placebo effect. It refers to the reinforcement of a patient’s expectation to get well. It may thus be worth exploring how to optimize these behavioral effects of magnetic stimulation in patients with functional disorders.

It is unlikely that these results and their interpretation are biased by a particularity of the population. Hence, our patients are comparable with previous FMD patient groups reported in the literature (2, 11, 30, 31), with a mean age of 40–50 years at onset, a clear female predominance, and a predominance of dystonia and tremor. Psychiatric comorbidities were frequent, as previously reported in patients with FMDs: nearly two-thirds of our patients had anxiety disorders, sometimes associated with depression (3). A high proportion (64%) of our patients had experienced a traumatic life event. Although traumatic life events have been linked to psychogenic non-epileptic seizures (32–34), their relationship with FMDs is less well documented and more controversial (35, 36). This high prevalence of traumatic life events in our series raises the possibility of a pathogenic role in FMDs. Finally, 21 of our 33 patients were not in employment, and 15 of them were receiving illness-related financial allowances (long-term sick leave or disability allowance). This is in keeping with the low reported employment rate among patients with functional neurological symptoms (8), including FMDs (3), and further underlines the financial costs of FMDs for society.

On day 3, after two sessions of low-frequency repeated magnetic stimulation, a large majority of our patients (22/33) experienced a clear improvement, which largely persisted after 1 year of follow-up. As we have previously shown, the degree of improvement did not correlate with any baseline clinical parameters, including symptom duration (11). Thus, although this was not our primary objective, our results further support the therapeutic use of magnetic stimulation in patients with chronic FMDs.

Future studies should disentangle the respective contributions of suggestion and motor relearning mechanisms that may be at work in this strong and sustained therapeutic effect, by manipulating the corresponding factors.

## Ethics Statement

All subjects gave written informed consent in accordance with the Declaration of Helsinki. The study was approved by the local ethics committee (CPP-IdF-Paris 6, Pitié-Salpêtrière University Hospital).

## Author Contributions

BG: design of the work, analyses of data, interpretation of data, drafting the work and revising the work, and final approval. FM, CH, TM, and II: design of the work and acquisition of data, revision of draft, and final approval. LN: conception of the research project, revision of draft, and final approval. MV and ER: interpretation of data, revision of draft, and final approval. BD: design of the work and acquisition of data, interpretation of data, revision of draft, and final approval.

## Conflict of Interest Statement

The authors declare that the research was conducted in the absence of any commercial or financial relationships that could be construed as a potential conflict of interest.
