# Supplementary material for: Impact of Transcranial Magnetic Stimulation on Functional Movement Disorders: Cortical Modulation or a Behavioral Effect?
Source: Front Neurol. 2017 Jul 19;8:338. doi: 10.3389/fneur.2017.00338 (PMC5515822; doi:10.3389/fneur.2017.00338)
Supplement: Supplementary file 1 [file Data_Sheet_1.docx]

**Supplementary material**

**Modified Abnormal Involuntary Movements Scale adapted for psychogenic movement disorder**

**1. Facial and Oral Movements**

Muscles of facial Expression (e.g., movement of forehead, eyebrows, periorbital area, cheeks; include frowning, blinking, smiling, grimacing)

1. None, normal

2. Minimal

3. Mild

4. Moderate

5. Severe

**2. Extremity Movements** **Upper** (arms, wrists, hands, fingers).

Include choreic movements (i.e., rapid, objectively purposeless, irregular, spontaneous), athetoid movements (i.e., slow, irregular, complex, serpentine).

1. None, normal

2. Minimal

3. Mild

4. Moderate

5. Severe

**3. Extremity Movements** **Lower** (legs, knees, ankles, toes).

(e.g., lateral knee movement, foot taping, heel dropping, foot squirming, inversion and eversion of foot.)

1. None, normal

2. Minimal

3. Mild

4. Moderate

5. Severe

**4. Trunk Movements Neck, shoulders, hips** (e.g., rocking, twisting, squirming, pelvic gyrations)

1. None, normal

2. Minimal

3. Mild

4. Moderate

5. Severe

**5 Global judgments**

Severity of abnormal movements:

1. None, normal

2. Minimal

3. Mild

4. Moderate

5. Severe

**6. Disability Score from the BFM scale**

#### Walking

1. normal
2. slightly abnormal; hardly noticeable
3. moderately abnormal; obvious to naive observer
4. considerably abnormal
5. needs assistance to walk

6- wheelchair-bound

***Scoring Sheet***

| **Cassette n°** |  |  |  | **Date :** |  |  |  |  |  |  |
| --- | --- | --- | --- | --- | --- | --- | --- | --- | --- | --- |

***Modified AIMS***

|  | **Score**  **(1-5)** | **Predominant movement** (Resting, postural or action tremor)/ dystonia/ dyskinesia/ myoclonus/ chorea/ fixed posture) | **Comments** |
| --- | --- | --- | --- |
| Face | \|___\| |  |  |
| Right Arm | \|___\| |  |  |
| Left Arm | \|___\| |  |  |
| Trunk | \|___\| |  |  |
| Right Leg | \|___\| |  |  |
| Left Leg | \|___\| |  |  |
| Global judgement | \|___\| |  |  |
| **Total Score (7-35)** | **\|___\|** |  |  |

## Dystonia rating –Burke – Disability Scale

**Comments**

| **Walking (0-6)** | **\|___\|** |  |  |
| --- | --- | --- | --- |

***Total rating***

| **Total Score (7-41)** |  |
| --- | --- |
