# Supplementary material for: Impact of Transcranial Magnetic Stimulation on Functional Movement Disorders: Cortical Modulation or a Behavioral Effect?
Source: Front Neurol. 2017 Jul 19;8:338. doi: 10.3389/fneur.2017.00338 (PMC5515822; doi:10.3389/fneur.2017.00338)
Supplement: Supplementary file 2 [file Table_1.DOCX]

**Supplementary Table 1. Detailed characteristics of the patients’ group.**

Gender: F=Female, M=Male.

Current psychiatric comorbidities: A: Anxiety disorder; D: major depression.

Intervention group: T=Transcranial stimulation was administered first (day 1), R=Root stimulation was administered first.

Body region: UL= Upper Limb; LL=Lower Limb

| **Patient Number** | **Gender** | **Age at inclusion** | **Duration (years)** | **FMD score at baseline (/41)** | **Improvement at day 2 (%)** | **Final improvement (%)** | **HAD score (/42)** | **Current psychiatric comorbidities** | **intervention group (first treatment)** | **traumatic life events** | **Main Movement Disorder** | **Body Region** | **Number of relapses** |
| --- | --- | --- | --- | --- | --- | --- | --- | --- | --- | --- | --- | --- | --- |
| 1 | F | 18.8 | 4.8 | 12 | 20 | 100 | 3 | 0 | T | 0 | Tremor | 4 limbs | 0 |
| 2 | F | 19.3 | 1.3 | 12 | 40 | 80 | 2 | 0 | R | 0 | dystonia | Right UL | 0 |
| 3 | F | 72.1 | 11.8 | 15 | 37.5 | 75 | 25 | 0 | T | other | Tremor | whole body | 1 |
| 4 | F | 49.1 | 1.7 | 28 | 0 | 42.9 | 4 | A | R | rape | Tremor | 4 limbs | 5 |
| 5 | M | 35.1 | 0.3 | 30 | 13 | 82.6 | 9 | 0 | R | other | jerky dystonia | whole body | 3 |
| 6 | F | 21.9 | 3.9 | 22 | 100 | 100 | 12 | A | R | other | Tremor | Right UL +LL | 0 |
| 7 | F | 26.3 | 1.5 | 16 | 11.1 | 22.2 | 8 | A | R | rape | Tremor | Right UL +LL | 1 |
| 8 | F | 42.4 | 5.3 | 35 | 17.9 | 96.4 | 5 | 0 | R | other | stereotypies | whole body | 2 |
| 9 | F | 29.9 | 7.2 | 9 | 0 | -50 | 7 | 0 | R | other | myoclonus | head | 0 |
| 10 | F | 46.7 | 2.3 | 17 | 60 | 70 | 9 | A | T | 0 | dystonia | whole body | 0 |
| 11 | F | 51.3 | 18.4 | 28 | 23.8 | 23.8 | 10 | A+D | T | rape | dystonia | 4 limbs | 0 |
| 12 | F | 54.9 | 22.4 | 10 | 100 | 100 | 10 | A+D | T | rape | dystonia | whole body | 1 |
| 13 | F | 49.9 | 19.6 | 22 | 20 | 100 | 8 | A | R | rape | Tremor | whole body | 1 |
| 14 | F | 42.9 | 3.6 | 27 | 60 | 80 | 21 | 0 | T | other | Tremor | 4 limbs | 1 |
| 15 | M | 46.1 | 2.3 | 14 | 57.1 | 100 | 18 | A | T | other | jerky dystonia | left LL | 0 |
| 16 | M | 56.1 | 2.8 | 19 | 0 | 50 | 17 | A | T | other | Tremor | 4 limbs | 0 |
| 17 | M | 56.7 | 1.6 | 23 | 0 | 0 | 18 | A+D | T | 0 | dystonia | whole body | 0 |
| 18 | F | 45.2 | 2.9 | 41 | 100 | 100 | 3 | A+D | R | 0 | dystonia | whole body | 0 |
| 19 | F | 61.1 | 10.5 | 11 | 0 | 50 | 21 | A | T | other | Tremor | right UL | 0 |
| 20 | M | 26.7 | 2.5 | 9 | 100 | 0 | 13 | 0 | T | other | dystonia | head + trunk | 0 |
| 21 | F | 37.9 | 1.3 | 22 | 20 | 26.7 | 5 | 0 | T | rape | dystonia | whole body | 1 |
| 22 | F | 28.9 | 1.7 | 19 | 33.3 | 66.7 | 25 | A | T | other | dystonia | LL | 0 |
| 23 | F | 37.5 | 6.2 | 24 | 100 | 100 | 8 | A+D | T | 0 | parkinsonism | 4 limbs | 0 |
| 24 | M | 60.7 | 2.9 | 19 | 33.3 | 33.3 | 11 | A+D | R | rape | jerky dystonia | 4 limbs | 0 |
| 25 | F | 72.8 | 22.4 | 8 | 100 | 100 | 26 | A+D | R | 0 | stereotypies | tongue | 0 |
| 26 | F | 28.7 | 1,0 | 29 | 72.7 | 100 | 17 | A | T | 0 | Tremor | 4 limbs | 1 |
| 27 | F | 68.5 | 1.3 | 22 | 0 | 0 | 35 | 0 | R | other | jerky dystonia | whole body | 0 |
| 28 | F | 53.6 | 18.6 | 16 | 55.6 | 100 | 12 | 0 | R | 0 | myoclonus | right UL + LL | 1 |
| 29 | F | 73.7 | 30.1 | 31 | 29.2 | 79.2 | 5 | A | R | rape | Tremor | whole body | 0 |
| 30 | F | 16.6 | 3.9 | 18 | 0 | 18.2 | 16 | 0 | R | other | dystonia | 4 limbs | 0 |
| 31 | M | 48.9 | 25.4 | 10 | 100 | 66.7 | 15 | A | T | 0 | Tremor | left LL | 0 |
| 32 | F | 20 | 0.5 | 18 | 27.3 | 27.3 | 16 | A | R | 0 | Tremor | whole body | 0 |
| 33 | F | 20.5 | 2.8 | 24 | 11.8 | 58.8 | 7 | 0 | T | 0 | dystonia | right LL + UL | 1 |
