# Supplementary material for: Impact of Transcranial Magnetic Stimulation on Functional Movement Disorders: Cortical Modulation or a Behavioral Effect?
Source: Front Neurol. 2017 Jul 19;8:338. doi: 10.3389/fneur.2017.00338 (PMC5515822; doi:10.3389/fneur.2017.00338)
Supplement: Supplementary file 3 [file Table_2.DOCX]

**Supplementary table 2. Improvement Rate according to clinical presentation.**

Clinical presentation:

other= Myoclonus, Stereotypies, and Parkinsonism.

Dystonia= dystonia and jerky dystonia

| **Clinical presentation** | **Number of patients** | **Improvement rate % (Median)** | **Inter Quartile Range** | **Improvement rate % (Mean)** | **Statistics (Kruskal Wallis)** |
| --- | --- | --- | --- | --- | --- |
| Tremor | 13 | 75 | 50-100 | 68.7 | p=0.24 |
| Dystonia | 15 | 58.8 | 18.2-82.6 | 50 |  |
| Other | 5 | 70 | 96.4-100 | 69.2 |  |
